# COVID-19 and nurse faculty caring: A meta-synthesis

**DOI:** 10.1016/j.heliyon.2024.e28472

**Published:** 2024-03-27

**Authors:** Nompumelelo Ntshingila, Charlene Downing, Dikomo Dorcas Rathaba, Marie Hastings-Tolsma

**Affiliations:** a University of Johannesburg Department of Nursing, P.O. Box 524, Auckland Park 2006, USA; b Baylor University Louise Herrington School of Nursing, 333 N. Washington St.Dallas, Texas, 75246, USA

## 1 Introduction

The COVID-19 virus infected approximately 600 million individuals globally, resulting in over six million deaths [1]. Nurses and midwives, including faculty members, experienced significant distress in the struggle to care for those infected and educate healthcare providers. In a profession where caring is a fundamental tenet [2], the nurse faculty experienced clear conflict during the pandemic related to student engagement and the subsequent provision of quality patient care. Uncertainty was also experienced as the faculty looked to protect their personal health [3].

COVID-19 had a negative impact on faculty well-being [4], taxing resilience abilities [5] and forcing faculty to engage in teaching strategies that often required a dramatic departure from their usual educational approaches [6]. Faculty have been burdened with providing discipline-specific content within the context of the clinical setting in a manner thrust upon them. The need for a caring faculty presence during teaching-learning also required consideration during the pandemic [7]. Forced to reflect on pedagogy and reconstruct approaches that would reduce students' exposure to COVID-19, caring solutions that minimised disruptions in learning were vital. An examination of faculty burdens while working to meet students’ learning needs was crucial in understanding their challenges and how caring was embraced.

## 2 Background

In 2019, a cluster of respiratory infections with unclear origins emerged in China [1]. Severe Acute Respiratory Syndrome Coronavirus 2 (SARS-CoV-2), commonly called COVID-19, quickly spread worldwide [8]. The resulting pandemic significantly impacted health services and healthcare providers’ training. Educators had to quickly change teaching methods, largely to an online format, which required a revision of instructional strategies [9].

For nursing faculties, the pandemic often required novel approaches to content presentation, which were time-consuming and demanding [10]. Although the pandemic wanes, educators continue to face challenges in meeting students' learning needs [11] while addressing personal safety concerns and interpersonal engagement [12]. Further, the shift to online education mandated greater resources, an ability to adapt to role changes, efficient technological and pedagogical management systems, and mentorship [13]. Other challenges related to students' access to technology, unpreparedness for new teaching and learning environments, maintaining online test security, and accessible interactive technology (IT). These challenges contributed to faculties’ anxiety about effective teaching in a practice discipline [14] and meeting regulatory requirements [15].

Those working with COVID-19 patients reported severe anxiety related to patient deaths, distress in delivering bad news, fear of contamination, staff and equipment shortages, ethical challenges, exhaustion and burnout, depersonalisation, post-traumatic stress disorder, worry about others embracing prevention measures, reduced social support, and anxiety that their own health was not prioritised [[16], [17], [18], [19], [20], [21]]. Such experiences were especially acute for novice healthcare providers who often lacked support and confidence in transitioning into practice, compounded by the pandemic [22]. Further, providers' concerns about quality care impacted the nurse-patient relationship and required creative strategies to mitigate stressors [23]. In short, healthcare providers were overwhelmed and exhausted [24]. Despite concerns about the media's underrepresentation of nurses' experiences with COVID 19.

[25], there has been even less research on faculties' experiences in preparing students. There is a need to clarify how faculty members embrace caring in the promotion of students’ professional development. Such an examination has the potential to alter the education landscape with a long-term impact on the nursing workforce. Ultimately, the challenges educators faced during the pandemic are clear, yet solutions are murky. A review of existing research that explored the caring strategies nurse faculties implemented may provide high-quality evidence capable of altering faculty-student-clinical partner engagements during pandemics.

## 3 The review

### 3.1 Objective

Common, important, and fundamental elements that may be used to faculty-student engagement were identified after a thorough review of research exploring nursing faculties' caring strategies in the face of the COVID-19 pandemic. Such distillation offered a richer understanding of faculties’ caring strategies in working with students than was available from a review of distinct qualitative studies. A synthesis of the most important aspects of faculty caring throughout the pandemic was facilitated by this expansive interpretative approach. The findings can be utilized to inform future research directions and guide faculty members in order to create a more comprehensive narrative.

### 3.2 Design

The analysis was guided by Noblit and Hare's [26] meta-ethnographic approach. The approach comprises seven overlapping phases frequently used as a guide for evaluating interpretive works [27]. New knowledge was synthesized using a comparative, inductive, interpretive approach, where selected works were translated into one another.

#### 3.2.1 Search strategy

Meta-synthesis requires an in-depth, unbiased, and replicable search of various sources to identify suitable studies. Bias was reduced through consultation with an academic librarian and further reduced through a broad review of electronic databases; seven (7) databases were examined [28]. A title and abstract search followed to identify relevant studies consistent with the inclusion criteria.

#### 3.2.2 Inclusion criteria

##### 3.2.2.1 Sample

Studies were included where the following criterion were met: a) qualitative method used, b) focus on COVID-19 and academic nurse faculty caring, and c) nursing students were the population of faculty interest. Research was included if mixed-method or qualitative and clearly stated the methodology. Studies also needed to be in English and with full text and published between December 2019 and December 2023. The starting point for including published studies was 2019 as this was when COVID-19 was first reported [1]. Use of these inclusion criteria ensured the relevance of all works and provided researchers with a cluster of studies that could be synthesized in our research [29]. All studies were excluded when qualitative methodology was not reported, work was non-English, were published prior to 2019, or were reviews, opinion pieces, concept analyses, dissertations, or theses.

#### 3.2.3 Study selection

**Fig. 1:**
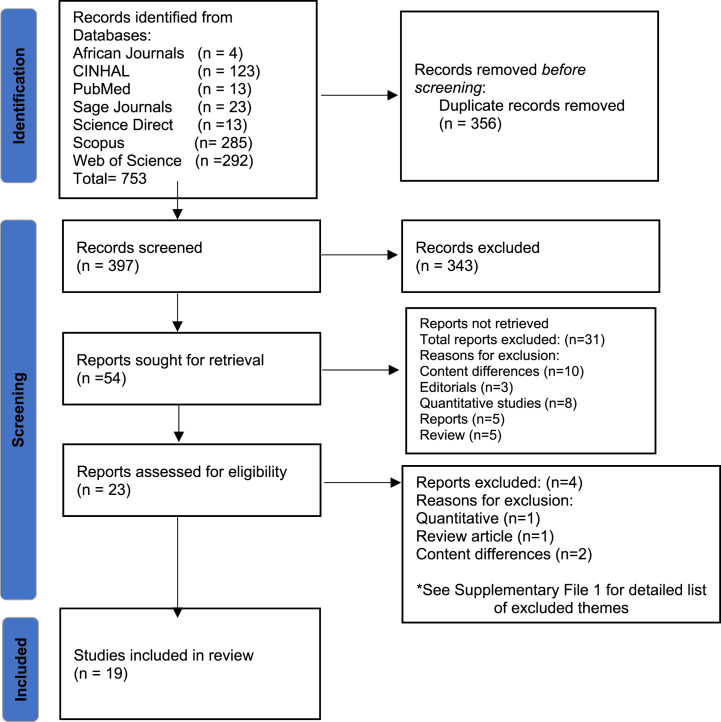
PRISMA Flow Diagram for Search strategy and selection of studies related to COVID-19 and nurse faculty caring (adapted from Page et al., 2021).

A librarian searched the databases using identified search terms. The search was augmented by back-tracking references to provide depth [30]. Three hundred and fifty six (356) duplicates were removed. The researchers then screened titles and abstracts against the inclusion criteria. Potentially relevant full-text papers were then retrieved and independently assessed by three of the researchers for inclusion based on identified criteria. The initial exclusion consisted of 343 articles (See Fig. 1 for all screened, excluded, and selected articles.).

The study followed a meta-synthesis method, which is a process of identifying and collating qualitative studies and synthesising the findings from the studies [31]. This method involved substantial dialogue with an academic librarian who guided investigators in determination of academic databases that needed to be searched [28]. Databases that were searched included African Journals (formerly SAePublications), Cumulative Index to Nursing and Allied Health Literature (CINHAL), PubMed, Sage Journals, Science Direct, Scopus and Web of Science. Boolean operators (AND, OR, NOT) and the keywords “nursing faculty and covid” were utilized in conducting the searches.

The use of reference databases for a meta-synthesis enables the search functions of each database to be used optimally when searched individually [32]. The meta-synthesis process also entailed a systematic gathering and analysis of qualitative studies. The researchers were able to acquire an understanding of the studies and interpret the findings of the particular topic in question [33].

The search for qualitative studies required that the focus be on nursing faculty, nursing academia or nursing academics, caring and Covid from January 2019–December 2023. The search of databases resulted in 19 articles that were included. Table 1, Table 2 provide a detailed outline of the examined studies, their contribution to findings, and a quality appraisal of the studies. Articles were excluded based on the following exclusion criteria and themes.

⁃ Content differences (n = 12)
⁃ Editorials (n = 3)
⁃ Quantitative studies (n = 9)
⁃ Reports (n = 5)
⁃ Reviews (n = 6)

**Table 1:** Description of included articles (N = 19).

Study/Setting	Scope/Purpose	Design, Methods	Sampling Strategy, Participants	Analytic Strategy
Bdair (2021) Ghad International Colleges for Applied Medical Sciences., Saudi Arabia.	To explore the nursing students‘ and faculty members ‘perspectives of online learn-ingduring the COVID-19 era.	A qualitative study was conducted using a descriptive phenomenology method.	N = 10 Nursing faculty members	Thematically analyzed using Spielberg's three-step process: intuiting, analyzing, and describing
Hopkins, Spadaro, Hoh, Singh, & Doas, (2022). USA.	To explore faculty experiences transitioning to a virtual Doctor of Nursing Practice residency.	Qualitative descriptive design	N = 11 nursing faculty with experience of virtual Doctor of Nursing Practice residency	Content analysis
Iheduru-Anderson & Foley (2021) USA.	To explore the experiences of associate degree nurse faculty who transitioned to online teaching during the early months of the COVID-19 pandemic in the United States	A qualitative, descriptive phenomenological design	N = 41 nursing faculty	Inductive thematic analysis approach described by Braun & Clarke)
Ignacio, Chen & Roy (2022), Singapore.	To describe and evaluate, through focus group discussions, a virtual collaborative learning activity implemented to assist first-year undergraduate nursing students to develop cognitive integration in a module consisting of pathophysiology, pharmacology, and nursing practice	A qualitative study using focus group discussions	N = 4 nursing practice faculty	Thematic analysis
Kunaviktikul et al. (2022). Southeast Asia.	To explore the experiences of nursing students and faculty members as related to online education during the COVID-19 pandemic	A descriptive qualitative design using photovoice	N = 28 nursing faculty members who participated in online education during the COVID-19 pandemic	Thematic analysis
Lynn & Ward-Smith (2021). USA.	To describe faculty experience of transitioning from face-to-face to an online format.	Qualitative descriptive	N = 10 undergraduate and graduate nursing faculty with web-based	Colaizzi's phenomenological analysis technique
Martin et al. (2023). USA.	To assess the impact of institutional, academic, and demographic characteristics on prelicensure nursing students' academic, initial postgraduation, and early career outcomes during the COVID-19 pandemic.	A qualitative, hermeneutic phenomenological methodological approach using focus groups	Four focus groups with nursing faculty; N = 26	Thematic analysis
McKay et al. (2022). USA.	To describe the experiences of baccalaureate nursing clinical faculty who transitioned from in-person clinical to emergency remote clinical teaching during the COVID-19 pandemic in spring 2020.	Qualitative descriptive	N = 19 clinical nursing faculty were interviewed	Conventional content analysis
Mokoena-de Beer et al. (2022). South Africa	To explore and describe nurse lecturers' experiences with online teaching during the COVID-19 pandemic at a public university.	Qualitative exploratory	N = 6 lecturers teaching in the undergraduate and graduate nursing programs	Babbie's content analysis
Moradi et al. (2022), Iran.	The aim was to explain the strengths and weaknesses of asynchronous e-learning in nursing education throughout the COVID-19 crisis.	Qualitative descriptive study	N = 14 nursing faculty members	Content analysis approach by Graneheim and Lundman
Murphy et al. (2022). USA.	To explore the perspectives and experiences of family nurse practitioner faculty and family nurse practitioner nursing students participating in flipped learning during the COVID-19 pandemic.	Insider AR qualitative study design	N = 4 graduate faculty in the family nurse practitioner program	Charmaz constructivist grounded theory data analysis methods
Nabolsi et al. (2021). Jordan.	To explore the first experience of nursing faculty members with online distant education (ODE) within the context of COVID-19 national curfew.	Qualitative descriptive guided by a phenomenological approach	15 faculty members working at two universities offers a nursing baccalaureate program.	Qualitative analysis, using Colaizzi's method
Ridgway et al. (2022). Australia.	To describe postgraduate maternal, child and family health nurse educators' perceptions of COVID-19 impacts on student knowledge of theory and practice, and lessons learned through their responses.	Qualitative, descriptive	semi-structured, videorecorded interviews of 6 nursing educators. Primary research question:	Braun and Clarke thematic analysis
Sacco & Kelly (2021). USA.	To describe nursing faculty experiences during COVID-19 pandemic	Qualitative, descriptive	Faculty (N = 49) teaching didactic course(s). Data collection online (Qualtrics) with one open-ended question:	Braun and Clarke thematic analysis
Sessions et al. (2022). USA.	To gain an understanding of the experiences of nurse educators during the COVID-19 crisis.	Qualitative description	N = 27 nurse educators employed in Maryland	Content analysis
Shindjabuluka et al. (2022). Namibia.	The purpose of the study was to explore how COVID-19 could serve as an enabler for the enhancement of online learning and teaching skills for nurse educators at the University of Namibia with specific emphasis on prospects and challenges	Qualitative explorative, descriptive and contextual research design	N = 18 nurse educators	Braun qualitative thematic analysis
Smith, Chen & Warner-Stidham, (2021).USA.	This study examined perceptions of online teaching effectiveness from nursing faculty and student perspectives.	A qualitative descriptive design was used to analyze data obtained from focus group interview sessions.	Nurse faculty (N = 15)	Thematic content analysis
Watson, (2023). USA.	The purpose of this study was to determine pre-licensure baccalaureate nursing student and nursing faculty perceptions of the effectiveness of various online teaching modalities.	A mixed methods study was used. Focused on the qualitative focus group with the nursing faculty members	Nursing faculty members N = 7 participated in focus groups	Grounded theory content analysis and Quirkos data analysis software
Wynter et al. (2022). Australia.	To assess the impact of the COVID-19 pandemic on nursing and midwifery educators across four large, multisite Australian health services.	Qualitative descriptive	All nursing and midwifery educators from public health services in Melbourne, Victoria (n = 3) and Adelaide, South Australia (n = 1)	Braun and Clarke Reflexive thematic analysis

**Table 2:** Summary of articles examined, contribution to findings, and quality appraisal (N = 19).

	Author/s, year, country	Purpose/Objectives	Key findings	Notes related to findings	Contribution to themes	QARI Score
1.	Bdair (2021) Ghad International Colleges for Applied Medical Sciences, SaudiArabia	To explore the nursing students ‘and faculty members ‘perspectives of onlinelearn-ingduringtheCOVID-19era.	Data were classified according to the three main studied domains: 1. advantages, 2. challenges, and 3. recommendations’	Faculty and students	1,2	8
2.	Hopkins, Spadaro, Hoh, Singh, & Doas (2022). USA.	To explore faculty experiences transitioning to a virtual Doctor of Nursing Practice residency.	1. Essence of residency was missing. 2. Virtual residency was an acceptable alternative.	Need to promote a relaxed scholarly environment, and uphold academic standards.	1,2	7
3.	Iheduru-Anderson & Foley (2021) USA.	Descriptive phenomenology was used to explore the experiences of associate degree nurse faculty who transitioned to online teaching during the early months of the COVID-19 pandemic	1. stressful/overwhelming, 2. feeling emotionally and physically exhausted, 3. support, 4. new knowledge and growth under pressure, 5. new opportunities for nursing education, and leadership in times of crisis.	To prevent the worsening of the nurse faculty shortage and help educate nurses for the future, nurse educators must be supported and recognised for their work during this period and beyond	1,2	8
4.	Ignacio, Chen & Roy (2022), Singapore.	To describe and evaluate, through focus group discussions, a virtual collaborative learning activity implemented to assist first year undergraduate nursing students to develop cognitive integration in a module consisting of pathophysiology, pharmacology, and nursing practice	Three themes also emerged from the focus group discussion scripts of faculty participants: 1. learning to effectively manage, 2. facing engagement constraints, and 3. achieving integration. These themes were further sectioned into salient subthemes	With better planning directed at addressing the learners' needs and the faculty's capabilities and readiness for online learning pedagogies, and with a strong institutional support to help mitigate the identified constraints of virtual collaborative learning, students and faculty will benefit	2	9
5.	Kunaviktikul et al. (2022). Southeast Asia.	To explore the experiences of nursing students and faculty members as related to online education during the COVID-19 pandemic	1. Psychological roadblocks to online education 2. Developing resilience despite adversities 3. Online education: What worked and what did not.	Highlighted the need for online psychological care and support to help participants better cope with anxiety, loneliness and home learning/teaching challenges.	1,2	8.3
6.	Lynn & Ward-Smith (2021). USA.	To describe faculty experience of transitioning from face-to-face to an online format.	1. How was your teaching altered in response to COVID-19? 2. What physical and/or psychological changes did you noticed in your students since the course format was altered? 3. In your role as faculty and nurse, what interventions did you develop and implement that assisted students to remain on track to complete the course requirements? 4. Descriptions of interventions believed to be wanted, as requested by your students	Communication with openness, coupled with patience and flexibility among faculty and students allowed courses to continue, assignments to be completed, and course grades to be awarded without delay.	1,2	8
7.	Martin et al. (2023), USA.	To assess the impact of institutional, academic, and demographic characteristics on prelicensure nursing students' academic, initial postgraduation, and early career outcomes during the COVID-19 pandemic.	1. Humanness of nursing education 2. Fostering salience through turmoil 3. New horizons for healthcare	Participants learned to challenge assumptions in nursing education during the pandemic, and through those experiences, they brought new perspectives to what was important to faculty	1,2	8
8.	McKay et al. (2022). USA.	To describe the experiences of baccalaureate nursing clinical faculty who transitioned from in-person clinical to emergency remote clinical teaching during the COVID-19 pandemic inspring 2020.	1. Transition 2. Collaboration and support, 3. The joy of teaching 4. Authentic professional experience 5. Stress of the moment	Faculty had to be innovative and work quickly to create appropriate and meaningful online clinical experiences. Collaboration and support from administration, IT, and faculty colleagues were identified by study participants as instrumental to the success of emergency remote clinical teaching.	1,2	8
9.	Mokoena-de Beer et al. (2022), South Africa.	To explore and describe nurse lecturers' experiences with online teaching during the COVID-19 pandemic at a public university.	1. Challenges related to the learner management system 2. Challenges related to competency 3. Factors out of the span of control of the lecturer 4. Indirect benefits of online teaching 5. Recommendations to facilitate the smooth delivery of online teaching.	Nurse lecturers experienced challenges when teaching online, resulting in frustrations and discomfort. Need for training and support to enhance online teaching and learning.	1,2	7.3
10.	Moradi et al. (2022), Iran	The aim was to explain the strengths and weaknesses of asynchronous e-learning in nursing education throughout the COVID-19 crisis.	1. Theme 1: Low quality of educational content 2. Theme 2: Cold and soulless education 3. Theme 3: Low efficiency in clinical education 4. Theme 4: Insufficiency in the educational assessment process	Asynchronous e-learning was shown to have various weaknesses in nursing theoretical and clinical education. However, the most striking strengths of this method in the COVID-19 pandemic were found to be the protection of the safety and health of individuals, followed by the maintenance of academic activities and education	1	8
11.	Murphy et al. (2022), USA.	To explore the perspectives and experiences of family nurse practitioner faculty and family nurse practitioner nursing students participating in flipped learning during the COVID-19 pandemic.	1. Flipped learning (FL) is a significant improvement that allows for continual student assessment and feedback. 2. Meaningful point values for in-class activities supports pre-class preparation.	Faculty preferred FL over traditional teaching approaches. The flipped classroom model enables instruction outside of the classroom that promotes student preparedness, while in-class student-centered active learning activities encourage critical thinking and cooperative learning.	1	6
12.	Nabolsi et al. (2021). Jordan.	To explore the first experience of nursing faculty members with online distant education (ODE) within the context of COVID-19 national curfew.	1. Resolving immediate reaction toward abrupt compulsory online teaching; fulfilling teaching responsibilities; managing the challenges of ODE 2. Struggling with available resources and capabilities 3. ODE defeated geographic and time boundaries, and interrupted personal time management: yet a new learning experience; insufficiency of ODE 4. Achieving clinical competencies and learning outcomes	This study informs higher education administration to invest in online education and to develop crisis response plans to strengthen efficient future response to similar crisis.	1,2	8.3
13.	Ridgway et al. (2022). Australia.	To describe nurse educators' perceptions of COVID-19 impacts on student knowledge of theory and practice, and lessons learned.	1. Grappling with COVID-safe teaching and assessment 2. Learning in a virtual community; 3. Clinical placement tensions.	All participants voiced struggles, opportunities and innovations. There was uncertainty, increased flexibility, change opportunities, and new ways of connecting with family and others.	2	8
14.	Sacco & Kelly (2021). USA.	To describe nursing faculty experiences during COVID-19 pandemic	1. University- or administration-related issues 2. Increased workload and decreased resources 3. Faculty stress from uncertainty and the intersection of work and life 4. Student's educational experience 5. Faculty commitment and positive experiences 6. Nursing faculty and COVID-19 in the context of the current advocacy movements	Nursing faculty will need ongoing professional development for faculty unfamiliar with the online and virtual learning environment is essential. Nursing faculty may also benefit from strategies that foster resilience.	1,2	8
15.	Sessions et al. (2022). USA.	To gain an understanding of the experiences of nurse educators during the COVID-19 crisis.	1. Uncertainty within pandemic ambiguity, 2. Prioritizing pedagogy, 3. Professional commitment.	Need for increased administrative support to hone their craft as nurse educators in online environments. Online pedagogies must include ways to support students' emotional well-being and the development of clinical judgment.	1,2	9
16.	Shindjabuluka et al. (2022). Namibia.	The purpose of the study was to explore how COVID-19 could serve as an enabler for the enhancement of online learning and teaching skills for nurse educators at the University of Namibia with specific emphasis on prospects and challenges	1. Nurse educators' experiences of online learning and teaching 2. COVID-19 as an enabler for enhancing online learning and teaching skills 3. Strategies to sustain online teaching and learning effectiveness	Online skills training should be provided to nurse educators in advance, ICT staff should maintain the IT infrastructure such that it will effectively support online learning and teaching and nurse educators should be provided with new well-functioning computers with the latest software to support their online teaching.	2	9
17.	Smith, Chen & Warner-Stidham, (2021).USA.	This study examined perceptions of online teaching effectiveness from nursing faculty and student perspectives.	1. Factors influencing teaching effectiveness 2. Faculty characteristics and teaching strategies 3. Frustrations related to online. 4. Disconnection between faculty and student expectations 5. Establishing clarity around expectations 6. Advice for effective online TE (teaching effectiveness) 7. Faculty needs specific to online TE	Identifying the characteristics of online teaching effectiveness provides the foundation for establishing tangible constructs and robust evaluation, broadening the impact on learning outcomes, faculty development, and educational practice.	1,2	8
18.	Watson, (2023). USA.	The purpose of this study was to determine pre-licensure baccalaureate nursing student and nursing faculty perceptions of the effectiveness of various online teaching modalities.	1. Optimization of technology 2. Faculty as entertainment 3. Collaborative nature of online/virtual learning experiences, and impact on class performance.	The results of this study will assist faculty in developing effective online and in-person instruction which will optimize the teaching/learning experience.	1,2	7.5
19.	Wynter et al. (2022). Australia.	To assess the impact of the COVID-19 pandemic on nursing and midwifery educators across four large, multisite Australian health services.	1. Occupational impacts 2. Psychological impact	Adjusting to providing education during a pandemic. Managing an increased workload. Concerns about inability to carry out usual activities. Importance of support at work. Managing own mental health. Difficulties supporting the mental health of health service staff.	1,2	8.6

A comprehensive list of excluded themes is provided in Supplementary File 1. The study explored qualitative studies that focused on the nurse or nursing faculty and caring during Covid; any other Covid-related publications on different themes could not be included as they are outside the scope of the study. Given the nature of the Covid environment and challenges experienced by the medical field, various studies relate to Covid but did not meet the study's inclusion criteria. Moreover, duplicates were excluded since the CINHAL database, part of the EBSCO collection of databases, allows searchers to exclude duplicates by clicking on the last page of the search results, which indicates that duplicates are removed. Furthermore, the new EBSCO Discovery Service removes duplicates by default when searching the database [34]. A detailed Preferred Reporting Items for Systematic Reviews and Meta-Analyses (PRISMA) flow diagram, which outlines information during the different stages of a systematic review or meta-synthesis [35] is included in Fig. 1, reflecting the retrieved results and final publications included in this study.

### 3.3 Quality appraisal

The Qualitative Assessment and Review Instrument (QARI) was used to appraise the articles’ quality [36]. The instrument, which comprises ten questions about congruency, was chosen because research has shown that it is more sensitive to validity issues than other widely used tools [37]. Each selected source was independently reviewed and scored by each of the three nursing researchers. Scores were then averaged, followed by a discussion, where necessary, until consensus was reached on the scoring. No study was excluded based on quality appraisal since the aim was to explore faculty caring during the pandemic with an extraction of the common, core features of the construct.

#### 3.3.1 Data abstraction, synthesis, relationships between studies, translating and synthesising work

The researchers followed various principles in creating the data abstraction and capturing the selected articles. The details included in the data abstraction sheet reflected the scope/purpose; design/method; sampling strategy/participants; and analytic strategy. Details of each article's contribution to the theme were also captured on the datasheet as capture in Table 2. Studies were independently reviewed by three of the researchers to determine themes, subthemes, metaphors, and relationships. Three researchers have doctoral degrees in nursing, and one has a master's degree in business management with a specialisation in information and knowledge management and is a Faculty of Health Sciences librarian. Multiple discussions were then held to review the findings, and consensus was reached on the translation and synthesis of themes and subthemes, supported by quotes. Where consensus was not reached, the researchers reconsidered the themes, questioned each other, and remained open to broadening their perspectives and interpreting data until a final determination was made.

## Ethical approval

This meta-synthesis did not involve human research participants, and the research was deemed exempt from ethics committee review.

## 4 Results

### 4.1 Study characteristics

Of the 19 identified articles, the majority (n = 18) were published in nursing journals; only one appeared in in a non-nursing, interdisciplinary health journal. Ten of the studies were conducted in the USA, one in South Africa, one in sub-Saharan Africa, two in Australia, and one each in Iran, Jordan, Singapore, Saudi Arabia and Southeast Asia. Individual study samples ranged from 4 to 49 participants, with a total of 211 participants from diverse geographic settings. In the selected studies, data were collected using semi-structured interviews, photovoice, 1:1 interviews, focus groups, and both oral and written open-ended questions. Studies included the nurse faculty though the context varied, consistent with the broad range of academic engagement (see Table 1). Table 2 summarises the articles’ contribution to the findings, including their QARI scores, which ranged from 6 to 9 with a rater agreement of 88% and a final consensus agreement of 100%.

### 4.2 Central themes

Two themes were identified: physical and emotional challenges, and resources to support faculty communication. The themes were supported by 10 subthemes.

#### 4.2.1 Theme 1: physical and emotional challenges

The pandemic caused a major change in the teaching-learning environment for faculty members. Academic institutions rapidly applied measures to ensure the continuation of academic progress, which created emotional and physical challenges, and challenges in adapting to change among faculty members.

##### 4.2.1.1 Emotional challenges

Stressors were encountered by faculty, and the resulting distress while attempting to meet students’ learning needs exacerbated their struggle to enact a more demanding role in academia.

“Every human being has encountered additional stress and additional stressors in response to this global pandemic. My approach with the students has been to recognize this, respect that everyone … me included … are navigating new territory and to have grace, patience and be forgiving” [9].

“Last year nearly killed me!” [4].

“However, it’s almost like going through a war together. They’ve come through it at the other end feeling as a cohesive group” [38].

“I have not been a nurse educator for long. I have definitely not taught online before. I was already stressed before this happened, so my stress level was at an all-time high” [39].

“I had awful insomnia. I could not sleep, and even still now, I still have to take medication to help me go to sleep, which I did not have to do before. When I would lay down to go to sleep, there’s always something else I needed to do, so I couldn’t fall asleep because I was always thinking, oh, I need to do this. I’ve got to do that. The anxiety, it was very difficult to deal with” [39].

Participants expressed feelings of uncertainty and ambiguity. Rapid change, and the need to successfully deliver e-learning, added to the fragility of faculty skills. Some felt troubled by the swift turn of events [40], – that they had been ‘thrown in’ overnight [41], creating feelings of a “drastic move” compounded by unexpected changes [6,42].

##### 4.2.1.2 Physical challenges

Another struggle was maintaining a work-life balance. Support for students and the need to maintain the normality of life blurred home and work boundaries. A work-life balance is shaped by having boundaries between work and rest time. However, during the pandemic, the home was the office; separate entities were non-existent.

“… teaching online increased [faculty] workload significantly … far more 1-to-1 student meetings via video. I spent 50 to 60 percent more hours on my academic duties. Moreover, due to budget cuts and hiring freezes, there [was] no extra help” [4].

“There are no working hours … I work day and night, preparing materials … different than what I am used to in traditional education” [6].

“It was more time. It was challenging for me. It was a real challenge because I had to create some space in my busy schedule to get them to be here for face to face as well” [43].

“I have taught for over 15 years in nursing education, but the last four months have all but burned me out. It has become an obsession because you never seem to get away from the work, and there is no balance. I am up at 6 am and still at it at 9 pm. Students are stressed and need more reassurance. I just don’t have much more to give. I am retiring at the end of this” [39].

The pandemic also increased awareness of what resources were available to implement and sustain online efforts. Faculty in programmes where no online teaching strategies were in place before the COVID-19 pandemic faced particular difficulties. Vendor solicitations regarding product availability exacerbated confusion and stress for faculty who noted that “The information overload from vendors was frustrating and overwhelming” [39]. Further, during the implementation of e-learning, the need for dedicated equipment became apparent, with economic and geographic disparities increasingly obvious.

“We are in a very rural area, and so students and faculty had the same WIFI issues. Our college basically said, ‘Well, you can go sit in the McDonald’s parking lots and get their WIFI, or the library will give you WIFI if you go sit in their parking lot.’ Personally, I was pretty irritated with that … I didn’t feel like I had everything I needed to do my job” [39].

“One of the biggest problems we had was finding out that many of our students do not have adequate access to technology. Not only internet services, but computers. I was just shocked at the number of our students that did not even have a home computer. So that was very eye opening for us. I worried about the students being left behind” [39].

In addition to a shortage of resources for student learning, faculty simultaneously had to address the availability of resources for their own family.

“… the institution was not well prepared … [they] had to offer training to address this” [44].

“I have only one laptop at home, and four children at school [who] needed to use the laptop” [6].

##### 4.2.1.3 Challenges in adapting to change

Adapting to change was a constant pressure and restructuring the home and work environment created distress. Efforts were made to ensure that all academic activities were managed, promoting continuation and timely completion of training. The flood of changes attributed to COVID-19 forced faculty to maintain expected didactic and clinical instruction despite significant constraints in student engagement when using an online platform [45]. Such change mandated additional support to meet demands.

“A lot of the [clinic rooms] are quite small, so they couldn't fit in with social distancing an extra person, a student. And how do you supervise the students, say, filling in [the electronic health record], for example, and sit over the other side of the room while they're doing it?” [38].

#### 4.2.2 Theme 2: resources to provide support for faculty communication

##### 4.2.2.1 Human support

Faculty had a strong desire for training on the design and development of online modules. Such training must be offered in advance of any future pandemic likely to occur. Training went hand-in-hand with the need for institutional preparedness to support faculty and increased availability of IT support.

Where human support from peers, family, and administration was experienced, participants reported stronger positive relationships and interactions. However, the shift to online education also precipitated a philosophical conflict for many faculty.

“… nursing is an applied profession, and we provide care which implies touch. COVID-19 required … [a] switch from 100% hands-on to 100% hands-off … connectivity has been lost” [9].

“Nursing practice is the core of the nursing profession, without hands-on we cannot master the required skills, even the theoretical knowledge needs to be linked to practice, and this is a real challenge we faced during our experience” [46].

“… E-learning is more of a lecture and has a theoretical framework … it's not a good way to teach practical and clinical courses. Nursing is a major in which you need to learn practical skills. We seriously have fundamental problems in this regard” [47].

Faculty often felt vulnerable and isolated due to virtual learning and the absence of active classroom interaction. Some felt they were “teaching to myself,” “teaching a computer,” or that it was “like pulling teeth” to engage students who were notably needy [48]. The lack of 1:1 engagement and feelings of isolation lowered faculty motivation.

“This [online instruction] is very different from when we were physically [together] … [then] I could see their expressions … I feel lonely” [49].

“… uuhm teaching a clinical course through online it is not simple … it was not easy but we are trying but uuhm we do not even think that the students are getting it the way they should get it” [43].

“Lack of students' interaction with other students and with faculty is also reported as a challenge for both students and faculty members” [46].

Keeping students engaged and enthused about learning was difficult in the online environment and had some faculty feeling like “a one-woman-show” [44]. There was a sharp reduction in student interactions with a lack of personal connection. The change in pedagogy with a time-driven agenda caused feelings of vulnerability, isolation, being overwhelmed, and depersonalisation [40]. Such feelings were notably minimised where administrators and peers provided clear faculty support for mandated changes.

“I spent over 16 hours a day trying to get information into Blackboard. That was daunting” [39].

“Students (online) were quieter … not interacting like they would in the live setting. I like the personal contact with students – to get to know them” [40].

“[Team members] were important. Both because of the amount of work … [and] also because it helped get a bunch of perspectives, and I think that that helped it to be better …” [44].

“My colleagues have… been so incredibly important in my ability to cope with this … just knowing that there are people that have your back and are standing alongside you …” [48].

Technological competence was essential for the successful transition to online teaching [44]. Another important element was administrative support for effective course delivery. Such support was needed to address educators’ learning curve in mastering online teaching and overcoming various barriers (e.g., laptop access, stable internet connections, severe weather impact, and time zone differences). And finally, faculty needed administrative leadership providing a blueprint for moving forward.

“… designing the online course, I needed technical assistance that was not available. My university was not prepared to assign an IT technician during the lockdown of the country and with curfew it was difficult even to reach my office to get what I needed from my desktop. I had to work it out by collaborating with my colleagues” [6].

“We had … faculty [who] had never taught online or weren't as savvy online, and that was difficult. Getting that learning curve up and running too” [44].

“I did not imagine that I will be teaching online, I was not in any way prepared for it. I am what people call technologically challenged. My stress level was through the roof” [39].

“… [nursing leadership was in] short supply. [Some leadership had not shown] much consideration for the increased workload associated with the transition to remote learning” [39].

In addition, faculty understood their responsibility to provide activities that ensured student learning. However, IT services were often lacking, which faculty deemed essential. In instances where faculty had to resolve student-technology issues, frustrations ensued.

“Sometimes the Power Point Presentations were not compatible with smart phones and students were unable to view it. I had to send the presentation as a [word] document and students missed the audio explanation of the presentation” [6].

Where there was a lack of support from administrators and peers, and where faculty had little say in decisions, feelings of anger surfaced with negative perceptions of remote learning [6]. This was particularly true for part-time faculty [44]. Access to resources in support of remote learning and faculty engagement in decision-making processes ultimately enhanced successful faculty engagement. Support was noted to take many forms, including sharing of course materials, workload credit, financial compensation, or time off to prepare for the sudden transition to online education [48]. Such support reduced time and workload demands (e.g., course preparation) while recognising faculty efforts. In short, faculty identified technology support, resources, and administrative support as crucial for effective online teaching [50].

“… many, many hours trying to create new things, but I think that the reason why I survived with some sanity was because nursing faculty are very generous in sharing - not only just sharing wisdom but sharing tools” [44].

“We flipped our entire curriculum online in a week. Creating that was a lot of work … I think I worked 12-hour days, probably for two weeks, just to get it all done” [44].

Participants also recognised the need to prioritise professional engagement. The pandemic required faculty to be creative and innovative in their teaching approaches to ensure optimal online experiences. Online teaching strategies were often unknown to faculty and required focused time to execute; such efforts needed to be prioritised [51]. The teaching strategies included copyright-approved visuals, case studies, virtual scavenger hunts, guest speakers, flipped learning, and online patient videos with discussions and debriefings [42,52]. Prioritizing learning avenues for diverse educational approaches instilled faculty confidence and reduced dissatisfaction.

##### 4.2.2.2 The lack of communication

Participants reported a lack of communication during the pandemic that they attributed to minimal personal contact, which impacted student learning. Some students perceived online education as ‘teaching the self’ while requiring substantial funds [50]. Faculty recognised the need to engage students for the successful achievement of course objectives and acknowledged that it was difficult to estimate students' workload in the online environment.

“… [a] lack of opportunities to practice interpersonal, psychomotor, and communication skills handicap students in the clinical setting” [48].

“… that interpersonal content … I think they [students] sense there's something lacking, and I sense there’s something lacking” [6].

The inability to effectively communicate with students in the absence of classroom engagement also impacted effective collaboration. The online pedagogy lacked human connection, touch and caring [6], resulting in feelings of faculty isolation and reduced motivation to persevere with online teaching.

“[I felt like I was] teaching to myself, staring and talking to a black hole, and trying hard to teach a computer” [49].

Where connections were more tenuous, such as for adjunct or part-time faculty, the increased workload and distance from professional support systems exacerbated perceived communication deficiencies.

“I felt very isolated … I don't feel [like] adjunct faculty were very supported” [44].

“… some new faculty reported feeling like the ‘rug was pulled out from under’ them [39].

“… students needed continuous support, but I was tired of the number of calls and messages, with no time boundaries. I received calls at night that I had to deal with in such crisis … it was exhausting to me” [48].

“I found that [using videoconferencing software] opened up my world to connect to the students … on a level that I hadn't been able to do before” [38].

##### 4.2.2.3 Communication at different levels

Proactive interventions, such as regular communication with the class and students who seemed to be in distress, were necessary to facilitate instruction [9]. Some faculty used student group work to successfully facilitate communication [42]. Nonetheless, the pandemic hindered communication with students, altered collaborative interactions, and the proactive interventions necessary to sustain effective online teaching-learning [9,42]. Effective communication between facullty and students made it possible for classes to continue, assignments to be finished, and marks to be given on time.

“As a[n] educator, I think we need consistent feedback from our students. This feedback helps improve teaching skills online” [9].

Despite the need for clear communication with students, the process was taxing, and participants encouraged faculty to set time boundaries.

“Long hours were spent communicat[ing] with students … [but faculty need to set time limits in] responding to students' communications and requests … to allow time away from [faculty work]” [6].

Support for transparent, two-way intra- and interdisciplinary faculty communication was crucial to improve interpersonal relationships and collaborations, both internationally and within the home institution [4,6,48,51]. Support from colleagues and the broader community, as well as collaboration across teams, provided positive faculty experiences. Overall, sources of support included academic, peer, and administrative teams, as well as spouses and family members. Where support was noted, faculty were likely to perceive emergency remote clinical teaching as successful [39,44,50] and were more likely to develop the skills required to overcome academic challenges [49].

In summary, the themes and sub-themes identified across selected works demonstrated the enormous challenges the academic nurse faculty faced when navigating the COVID-19 pandemic. The synthesis of these works underscores the need for faculty support to minimize stress while ensuring students’ achievement of course objectives.

### 4.3 Interrelationship between themes

**Fig. 2:**
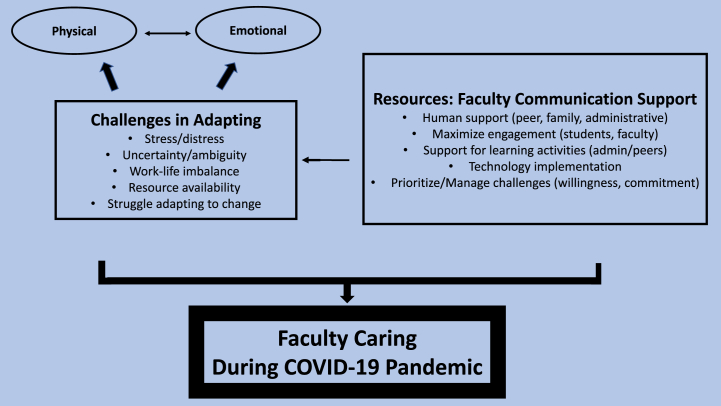
The interrelationship between identified themes and subthemes

Fig. 2 illustrates the interrelationship between identified themes and subthemes. Data analyses suggested that resources to support faculty communication were a foundational faculty characteristic and antecedent in adapting to the physical and emotional challenges triggered by the pandemic. Resources for effective communication were crucial to successfully manage roadblocks, collaborate with colleagues, and steer students to care through technological innovation. Where challenges were successfully addressed, faculty resilience was fostered despite the adversity created by the pandemic. The synthesis, generated from 19 studies with a total sample of 265 participants working in diverse geographic settings, demonstrated considerable similarities among faculty experiences in dealing with the fallout of COVID-19.

## 5 Discussion

The data from this study indicated the nurse faculty experienced physical and emotional challenges as well as challenges in adapting to change. The COVID-19 pandemic has resulted in considerable emotional strain and stress, culminating in sensations of burnout. These sensations might stem from a heightened workload, the introduction of unfamiliar technology, an increased necessity for guiding and supporting students, and the demands of faculty clinical practice [4]The nursing faculty experienced significant stress and anxiety amidst the pandemic as they acknowledged the potential repercussions of a global health crisis, yet initially grappled with understanding its full impact [48]. This ambiguity and uncertainty left the nursing faculty with a profound sense of the unfamiliar [53].

The results of this meta-synthesis support Nowel's [54] findings that the nurse faculty struggled to stay afloat during the COVID-19 pandemic. Educators faced overwhelming work pressures, extended hours, and unsustainable workloads, resulting in emotional and physical exhaustion. In a study conducted in the USA, numerous faculty members considered retiring or returning to bedside nursing because of the stress connected with the move to virtual teaching [39].

According to Boamah [55], the lack of balance between work and personal life, along with burnout among nursing faculty, can significantly impact key factors related to workplace retention in the long term. A work-life imbalance exacerbates the current nurse workforce shortages and creates a severe risk to faculty well-being, quality of life, and mental health difficulties [56]. In addition to their work-life imbalance, the nurse faculty faced resource constraints. Molato and Seholaro [57] assert that institutions of higher learning should be adequately equipped with essential resources, such as electronic devices, internet connectivity, and data access, to effectively utilize online learning modalities. Moreover, constant changes added to the faculty's stress as they adjusted to online learning. Changes could influence job satisfaction and, ultimately, the recruitment and retention of both newly hired and existing nurse faculty. The COVID-19 pandemic revealed significant weaknesses and inadequacies in nursing student and staff support programmes in institutions, particularly in many nursing colleges [39]. Gazza [58] similarly emphasised the importance of establishing measures that may improve working circumstances for nurse educators in the future.

Humanising caring for students during the pandemic created a deep understanding of the students' context and helped to create meaningful learning spaces for the students [59]. Caring for faculty members was also lost, since avenues for dropping into a colleague's office to chat and hallway conversations disappeared during the pandemic [60]. The shift from person-centered learning to online learning meant faculty had to embrace new skills within a limited time [61]. Technology acceptance among the nursing faculty is perceived as stressful within the spheres of usefulness and acceptance of technology [62]. This led to a continuation of online learning and the application of the latest technology in education; for example, online meetings, online assignments, online counselling, and a general sharing of information [61]. The nurse faculty extended their communication beyond the call of duty, conducting regular check-ins on students' well-being, and it was critical during class to determine overall well-being [48]. However, faculty often struggled to find ways to show caring towards students due to communication challenges [50].

## 6 Strengths and limitations

This research had several limitations. First, this meta-synthesis research may have missed seminal works. Specifically, theses were excluded as many were inaccessible or with prohibitive costs. Secondly, the researchers lacked direct access to the data generated in each individual study. Third, only 19 qualitative investigations were analyzed, though relatively few studies are sufficient for meta-synthesis [26] and may allow for greater depth of analysis [27]. Fourthly, a synthesis of studies was conducted by researchers different from those who carried out the original research, potentially leading to varied interpretations of the phenomenon [30]. Nevertheless, the meta-synthesis was performed by investigators within the same field, representing the most prevalent approach [63]. Fifth, no pilot work was conducted in referenced studies, which could impact the relevance and simplicity of the developed questions. Finally, studies were largely from the USA, with limited information regarding culture, which is a contextual factor that may have impacted findings.

## 7 Implications for nursing education

There are important concerns for teacher-learner engagement during a pandemic that should be considered in the academic milieu. Situations demanding a change in pedagogy require advanced preparation to promote a smooth, less stressful transition. Administrators should carefully consider the provision of resources to promote faculty competence in an online format, ongoing support for faculty, clear equipment availability, and compensation for faculty mandated to deliver instruction in an unfamiliar format. Similarly, faculty must also consider advanced planning for changes that would be mandated during a pandemic or other crisis. Faculty should consider skills necessary to promote flexible engagement with learners, approaches for supporting faculty, alternative plans to meet learner needs, and how self-care could be achieved. Compassionate care of self and others is a fundamental need among faculty and students [64,65] and, where absent, can contribute to high levels of stress, poor professional and family relationships, and compromised patient care. Table 3 details the implications for academic nursing faculty when faced with a pandemic/epidemic.

**Table 3:** Implications for nursing/midwifery education.

Faculty
Provide flexibility in meeting course objectives and deadlines while maintaining expectations.
Examine strategies that would keep students motivated and engaged in course work by meeting in advance with other faculty, students, and administrators.
Develop a central repository where course materials could be shared by faculty.
Consider strategies to minimize prolonged screen time in a course (e.g., recording lectures).
Consider developing case studies which require student engagement when online.
Develop assignments that require student teamwork and promote effective collaboration and communication.
Restructure student assignments/assessments to reduce faculty/student preparation time in advance of a pandemic/epidemic.
Where classroom based, consider strategies that promote student e-learning ability (e.g., engagement in 1–2 on-line course activities) in advance of a pandemic/epidemic.
Consult with IT to ensure presentation materials are formatted for varied devices (e.g., smart phone).
Consider meeting with clinical partners to ascertain effective student engagement and facility demands.
Limit time engagement in course activities to promote work-life balance and reduce stress.
Consider targeted time where stress-reducing practices are used (e.g., music, walking, massage).
Administration
Appoint a task force responsible for preparing a plan that supports faculty/students during a pandemic/epidemic.
Consider additional faculty compensation (e.g., financial, workload reduction) where rapid change in pedagogy is required.
Provide ongoing e-learning education/training in advance of a pandemic/epidemic.
Provide faculty with equipment (e.g., laptop computers) in advance of a pandemic/epidemic.
Consider establishing on-line support groups for faculty debriefing to reduce feelings of loneliness, isolation, and judgement.
Ensure IT support services are available 24/7 for faculty and student trouble shooting.
Assign every faculty member to a specific IT technician with clear contact information.
Consider assigning faculty mentors for those instructors in need of additional support, particularly part-time and adjunct faculty.
Assign co-faculty in any course where the faculty has had little experience with e-learning.
Prepare a back-up plan in the event of faculty/family impact by the pandemic/epidemic.
Reduce department requirements (e.g., meetings) to decrease faculty stress.
Assign a specific staff member to support each faculty (e.g., librarian to secure copyright permissions, contacting guest speakers, preparing materials for distribution to students).
Consider securing an instructional technology specialist to work with faculty in developing innovative and creative online course strategies.

Key: IT = instructional technology.

## 8 Conclusion

The pandemic created challenges and opportunities for academic nursing faculty to learn effective approaches in caring for students. While COVID-19 appears to be waning, another pandemic is probable [66], requiring faculty to be adaptable, resilient, and flexible. Moreover, online education will likely remain a fixed part of education. Online education, however utilized, must prepare a qualified nursing workforce in a timely fashion. The attainment of this objective requires faculty who are well-prepared and digitally literate, possessing the necessary knowledge and skills to effectively deliver online learning while demonstrating genuine care.

## Funding statements

The research study was financially supported by the 10.13039/501100006565University of Johannesburg.

## Data availability statement

Supplementary data to this article can be found in Supplementary file Appendix A.

## CRediT authorship contribution statement

**Nompumelelo Ntshingila:** Conceptualization, Methodology, Writing – original draft, Writing – review & editing. **Charlene Downing:** Conceptualization, Methodology, Writing – original draft, Writing – review & editing. **Dikomo Dorcas Rathaba:** Conceptualization, Methodology, Writing – original draft, Writing – review & editing. **Marie Hastings-Tolsma:** Conceptualization, Methodology, Writing – original draft, Writing – review & editing.

## Declaration of competing interest

The authors declare the following financial interests/personal relationships which may be considered as potential competing interests:Nompumelelo Ntshingila reports article publishing charges was provided by University of Johannesburg. If there are other authors, they declare that they have no known competing financial interests or personal relationships that could have appeared to influence the work reported in this paper.
