# Supplementary material for: COVID-19 and nurse faculty caring: A meta-synthesis
Source: Heliyon. 2024 Mar 27;10(7):e28472. doi: 10.1016/j.heliyon.2024.e28472 (PMC10990944; doi:10.1016/j.heliyon.2024.e28472)
Supplement: Multimedia component 1 [file mmc1.docx]

**Supplementary File 1: Exclusion criteria: Excluded Themes**

| - **Content differences (n=12)**   - Focus on students’ experiences (2)  - Focus on nursing program directors (1)  - Covid- 19 vaccine opinions (2)  - Professional nurses and Caring and Covid (1)  - Nurse educator students (1)  - Results focusing on educators in general and not nurse academics (5)   - **Editorials (n=3)** - **Quantitative studies (n=9)** - Descriptive correlational (1) - Cross-sectional study (2) - Quantitative (2) - Comparative study (1) - Mixed method (3) - **Reports (n=5)** - Reflections (1) - Report (4) - **Reviews (n=6)** - Scoping reviews (2) - Policy review (2) - Teaching and learning process review (1) - Experiential narrative review (1) |
| --- |
